# A Retrospective Review of Hospital-Based Data on Enteric Fever in India, 2014–2015

**DOI:** 10.1093/infdis/jiy502

**Published:** 2018-10-11

**Authors:** Dipika Sur, Caitlin Barkume, Bratati Mukhopadhyay, Kashmira Date, Nirmal Kumar Ganguly, Denise Garrett

**Affiliations:** 1 Translational Health Science and Technology Institute, Faridabad, India; 2 Sabin Vaccine Institute, Washington, D. C., Georgia; 3 Global Immunization Division, Centers for Disease Control and Prevention, Atlanta, Georgia

**Keywords:** Typhoid, paratyphoid, enteric fever, *Salmonella*, India

## Abstract

**Background:**

Enteric fever remains a threat to many countries with minimal access to clean water and poor sanitation infrastructure. As part of a multisite surveillance study, we conducted a retrospective review of records in 5 hospitals across India to gather evidence on the burden of enteric fever.

**Methods:**

We examined hospital records (laboratory and surgical registers) from 5 hospitals across India for laboratory-confirmed *Salmonella* Typhi or *Salmonella* Paratyphi cases and intestinal perforations from 2014–2015. Clinical data were obtained where available. For laboratory-confirmed infections, we compared differences in disease burden, age, sex, clinical presentation, and antimicrobial resistance.

**Results:**

Of 267536 blood cultures, 1418 (0.53%) were positive for *S.* Typhi or *S.* Paratyphi. Clinical data were available for 429 cases (72%); a higher proportion of participants with *S*. Typhi infection were hospitalized, compared with those with *S.* Paratyphi infection (44% vs 35%). We observed resistance to quinolones among 82% of isolates, with cases of cephalosporin resistance (1%) and macrolide resistance (9%) detected. Of 94 participants with intestinal perforations, 16 (17%) had a provisional, final, or laboratory-confirmed diagnosis of enteric fever.

**Discussion:**

Data show a moderate burden of enteric fever in India. Enteric fever data should be systematically collected to facilitate evidence-based decision-making by countries for typhoid conjugate vaccines.

Typhoid and paratyphoid fever (collectively known as enteric fever) is caused by the organisms *Salmonella* Typhi and *Salmonella* Paratyphi (serovars A, B, and C) and is a systemic disease that is endemic in many Asian countries where a large proportion of the population lacks access to safe water, sanitation, and hygiene infrastructure. *S.* Typhi and *S.* Paratyphi are estimated to cause nearly 12 million and 4 million annual cases of illness, respectively, and >153000 annual deaths, although accurate estimates are lacking and inconsistent because of the limited number of well-conducted studies [1, 2]. Although enteric fever is rare in industrialized countries, it remains an important and persistent public health problem in low-resource countries. In the countries most affected, however, barriers such as a lack of systematic public health reporting and laboratory infrastructure contribute to substantial knowledge gaps of the disease burden and presentation. In India, where pooled estimates have shown that nearly 10% of isolates from individuals with enteric fever have been identified as *S.* Typhi [3], there have only been 3 studies in 2 locations that have attempted to determine the incidence of enteric fever, and few hospital-based studies have been performed in recent years to understand the spectrum of disease [4–6]. Since a new typhoid conjugate vaccine (TCV; Typbar-TCV, Bharat Biotech International) has been recently recommended and prequalified by the World Health Organization (WHO) and included in the 2019–2020 funding window of Gavi, the Vaccine Alliance, additional data on the burden and clinical presentation of enteric fever in India is needed for decision-making on the introduction of the new vaccine and to understand its potential impact [7, 8].

While antimicrobial therapy is an effective treatment for enteric fever, an increasing rate of resistance to available antibiotics is resulting in higher morbidity, mortality, and cost of treatment [9–11]. The most commonly used diagnostic test is blood culture, which based on pooled estimates and has been shown to be only 61% sensitive [12]. Further, routine blood culture is not always available in low-resource settings, and physicians commonly rely on clinical symptoms, which are nonspecific from other febrile illnesses, to empirically treat enteric fever. This can lead to inappropriate treatment and, subsequently, increasing antimicrobial resistance. Results from a 12-year retrospective study in India showed an increase in reduced susceptibility to ciprofloxacin in *S.* Typhi isolates, which has also been recently shown in other South Asian countries, such as Nepal and Bangladesh [13–15]. Although recent patterns showed a decrease in multidrug-resistant isolates (ie, those resistant to ampicillin, chloramphenicol, and trimethoprim-sulfamethoxazole), emerging resistance to third-generation cephalosporins, the primary antibiotics of choice in recent years, has been increasingly seen in the South Asian continent, severely threatening treatment options while increasing treatment costs [16, 17].

A systematic review of studies on enteric fever in India revealed few community-based studies attempting to estimate typhoid and paratyphoid fever incidence and, in the last 10 years, only 7 hospital-based studies [3]. Since many recent studies in India have been characterized by a small sample size and were limited to single-center sites, additional data in India are needed to show burden of disease and provide evidence for the usefulness of TCVs [18, 19]. The absence of credible estimates of the disease burden in India has resulted in limited understanding of the impact of the disease and consequently hindered prevention and control efforts. Further, some studies have suggested a seasonal component to typhoid occurrence in India [5]. Elucidating the spectrum, temporality, and burden of disease will help inform typhoid prevention and control strategies through vaccines and other measures in countries where it is endemic.

We conducted this retrospective review to gather data on the enteric fever burden in India and to better explain the epidemiology and clinical profile of enteric fever cases across the country. As part of the Surveillance for Enteric fever in Asia Project (SEAP), this retrospective study aims to describe the clinical profile, severity, antimicrobial resistance, and outcomes of laboratory-confirmed enteric fever cases in India, using existing hospital data. This study also aims to review characteristics of intestinal perforation cases as a marker of disease severity.

## METHODS

### Study Design and Site Selection

We conducted a retrospective, cross-sectional study among patients with blood culture–confirmed *S*. Typhi or *S.* Paratyphi infection or intestinal perforation in different hospitals across India from 2014 to 2015. We selected hospitals that were secondary or tertiary-care facilities containing laboratory departments capable of diagnosing enteric fever with searchable electronic laboratory records. Five hospitals were identified and agreed to participate in the study: (1) the Postgraduate Institute of Medical Sciences (PGI), a mixed public-private tertiary-care hospital in Chandigarh with 1960 beds mainly serving an urban population; (2) Medanta Hospital (Medanta), a private tertiary-care hospital in Gurugram (previously known as Gurgaon), Haryana, with 1250 beds mainly serving an urban population; (3) Christian Medical College (CMC), a private tertiary-care hospital in Vellore, Tamil Nadu, with 2800 beds mainly serving an urban population; (4) Apollo Gleneagles Hospital (Apollo), a private tertiary-care hospital in Kolkata, West Bengal, with 750 beds mainly serving an urban population; and (5) Kasturba Medical College–Manipal University Hospital (KMC), a private tertiary-care hospital in Manipal, Karnataka, with around 2000 beds mainly serving a peri-urban population.

### Data Collection

Our data sources were electronic laboratory records and surgical department registers. The electronic laboratory records were searched to identify patients with laboratory confirmation of *S.* Typhi or *S.* Paratyphi infection by blood culture between January 2014 and December 2015. Data on demographic characteristics and hospital admission status of patients with laboratory-confirmed infection were initially extracted from the laboratory database. Study staff then used patient identification numbers of hospitalized cases to find inpatient medical charts.

Surgical department registers were searched to identify patients with an intestinal perforation between January 2014 and December 2015. Study staff used patient identification numbers of intestinal perforation cases to find inpatient medical charts.

For hospitalized patients with laboratory-confirmed infection or intestinal perforation who had available medical charts, staff abstracted laboratory results and clinical data (eg, duration of hospitalization, diagnoses, symptoms, and complications), using standard paper-based data collection forms. Data were entered into a database for analysis using Microsoft Access (Redmond, WA).

### Data Analysis

We conducted a descriptive analysis to compare burden differences, according to age and sex and antimicrobial resistance, between *S.* Typhi and *S.* Paratyphi infections, using the Pearson χ^2^ test, the Fisher exact test, or the nonparametric Wilcoxon rank sum test to determine statistical significance. We examined differences in age distribution by using nonparametric Kolmogorov-Smirnov 2-sample tests. We also reviewed the seasonality of case counts, by hospital. All statistical tests were 2-sided and considered statistically significant at a *P* value of <.05.

Data were analyzed using SAS, version 9.4 (Cary, NC).

### Ethical Considerations

The study protocols were reviewed and approved by the institutional ethics committees at the Translational Health Science and Technology Institute, the Postgraduate Institute of Medical Sciences, Medanta Hospital, Apollo Gleneagles Hospital, Kasturba Medical College–Manipal University Hospital, and the Institutional Review Board at Christian Medical College, Vellore. Identifying personal information was accessible only to evaluation staff at the sites; all data reported to investigators were deidentified.

## RESULTS

### Laboratory Cases

Among the 267536 blood cultures performed at the study hospitals during 2014 and 2015, 1418 (0.53%) were positive for *S.* Typhi or *S.* Paratyphi, including 1147 (81%) positive for *S.* Typhi and 271 (19%) positive for *S.* Paratyphi. The proportion of *S.* Typhi isolates was 86% at PGI, 82% at Apollo, 81% at CMC, 80% at Medanta, and 76% at KMC.

**Figure 1.**
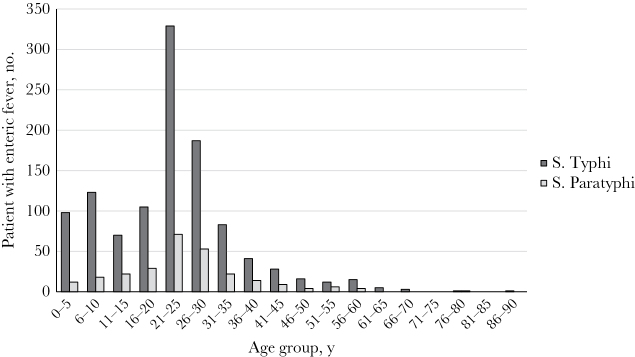
Age distribution, by 5-year age group, among 1382 patients with laboratory-confirmed enteric fever, by organism, all hospital sites, India, 2014–2015. : *S*. Paratyphi, *Salmonella enterica* subspecies *enterica* serovar Paratyphi; *S*. Typhi, *Salmonella enterica* subspecies *enterica* serovar Typhi.

Among the 1418 patients with laboratory-confirmed infection, 97% had information on age and sex available within laboratory records. The median age for all patients with laboratory-confirmed infection was 24 years (interquartile range [IQR], 18–30 years), and the sex of 54% was male (Table 1). The group aged 20–29 years had the highest percentage of both *S*. Typhi and *S*. Paratyphi infections (45%). While *S.* Paratyphi infections had a single-peaked age distribution around 25 years, *S*. Typhi cases peaked at 10 years and 25 years (Figure 1).

**Table 1.** Demographic Characteristics of Patients With Laboratory-Confirmed Enteric Fever, by Organism, All Hospital Sites, India, 2014–2015 (n = 1418)^b^

Characteristic	*S.* Typhi (n = 1147)	*S.* Paratyphi (n = 271)	Total (n = 1418)
Age, y^a^			
<2	15 (1)	1 (<1)	16 (1)
2–4	54 (5)	9 (3)	63 (4)
5–9^c^	123 (11)	18 (7)	141 (10)
10–19	180 (16)	45 (17)	225 (16)
20–29	513 (45)	122 (45)	635 (46)
30–39	144 (13)	44 (16)	188 (14)
40–49	45 (4)	14 (5)	59 (4)
≤50	43 (4)	12 (4)	55 (4)
Overall	24 (16–29)	24 (14–28)	24 (18–30)
Male sex^b^	585 (53)	153 (58)	738 (54)
Department			
Inpatient^c^	502 (44)	95 (35)	597 (42)
Outpatient	645 (56)	176 (65)	821 (58)

Data are no. (%) of patients or median (interquartile range).

Abbreviations: *S*. Paratyphi, *Salmonella enterica* subspecies *enterica* serovar Paratyphi; *S*. Typhi, *Salmonella enterica* subspecies *enterica* serovar Typhi.

^a^Data were missing for 36 patients.

^b^Data were missing for 39 patients.

^c^Significant difference between individuals infected with *S.* Typhi and those infected with *S.* Paratyphi (*P* < .05).

**Figure 2.**
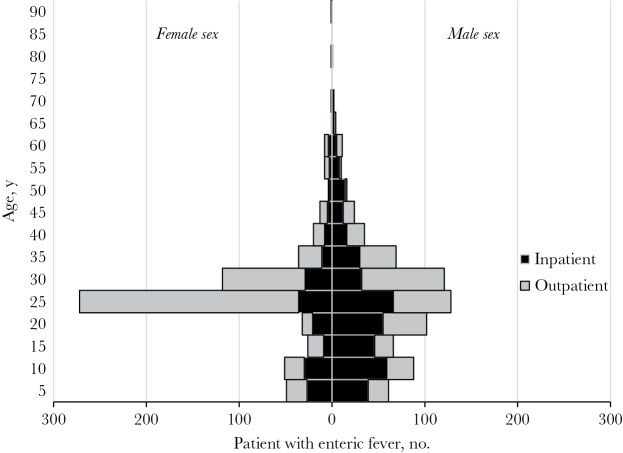
Age distribution of 1379 patients with laboratory-confirmed enteric fever, by sex, all hospital sites, India, 2014–2015.

In addition to differing by etiology (typhoid vs paratyphoid fever), the age distribution of confirmed infections also differed by sex (*P* < .001; Figure 2). Male patients had a broader age distribution curve (kurtosis = 0.79), shown by an interquartile range of 13–30 years, while female patients had a more tightly clustered age distribution (kurtosis = 3.88), shown by an interquartile range of 21–27 years.

**Figure 3.**
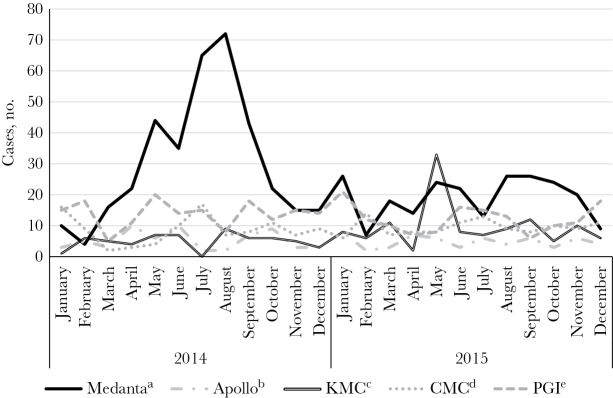
Cases of laboratory-confirmed enteric fever, by month and hospital site, India, 2014–2015 (n = 1418). ^a^Medanta Hospital, Gurugram, Haryana; ^b^Apollo Hospital, Kolkata, West Bengal; ^c^Kasturba Medical College–Manipal University Hospital, Manipal, Karnataka; ^d^Christian Medical College, Vellore, Tamil Nadu; ^e^Postgraduate Institute of Medical Sciences, Chandigarh.

We observed increases in the number of cases during the summer monsoon months (May–September) at Medanta in 2014; the number of cases peaked at 72 in August, which is 288% higher than the 2-year monthly average (25 cases during 2014–2015; Figure 3). We also observed an increase in cases at KMC in May 2015, compared with preceding and subsequent months, but observed no seasonal trends at Medanta in 2015 or at Apollo, CMC, and PGI for either year.

Of 1418 patients with laboratory-confirmed infection, 597 (42%) were hospitalized. A higher proportion of patients infected with *S*. Typhi were hospitalized, compared with patients infected with *S*. Paratyphi (44% vs 35%; *P* = .009; Table 1). Overall, male patients were statistically more likely to be admitted to the hospital for enteric fever infection than females (48% vs 29%; *P* < .001). Although this trend was present in virtually all age groups, it reached statistical significance among patients aged 11–15 years (70% vs 35%; *P* = .002) and 21–25 years (52% vs 13%; *P* < .0001; Figure 2).

### Clinical Data

Of the 597 hospitalized patients identified at the laboratories of all 5 hospitals, 429 (72%) had available medical charts, including 362 (84%) infected with *S.* Typhi and 67 (16%) infected with *S.* Paratyphi.

The most commonly reported symptoms among patients with hospitalized laboratory-confirmed infection included fever (in 97%), nausea/vomiting (in 50%), weakness/malaise (in 38%), headache (in 35%), abdominal pain (in 32%), diarrhea (in 29%), and cough (in 29%; Table 2). A higher proportion of patients infected with *S.* Typhi presented with gastrointestinal symptoms, compared with patients infected with *S.* Paratyphi (*P* = .027 for nausea/vomiting, and *P* = .011 for diarrhea). The median duration of fever at admission of enteric fever cases was 7 days (IQR, 5–14 days), and previous antibiotic use was reported among 21% of admitted patients with enteric fever. About 50% of enteric fever cases had a provisional diagnosis of enteric fever/typhoid, while fever of unknown origin was the second most common diagnosis (35%).

**Table 2.** Clinical Presentation of Hospitalized Patients With Laboratory-Confirmed Enteric Fever, by Organism, All Hospital Sites, India, 2014–2015

Variable	*S.* Typhi (n = 362)	*S.* Paratyphi (n = 67)	Total (n = 429)
Symptom at admission			
Fever	351 (97)	67 (100)	418 (97)
Nausea/vomiting^a^	188 (52)	25 (37)	213 (50)
Weakness/malaise	133 (37)	31 (46)	164 (38)
Headache	123 (34)	27 (40)	150 (35)
Abdominal pain	116 (32)	20 (30)	136 (32)
Diarrhea^a^	115 (32)	11 (16)	126 (29)
Cough	107 (30)	18 (27)	125 (29)
Skin rash	28 (8)	2 (3)	30 (7)
Blood in stool	15 (4)	0 (0)	15 (3)
Constipation	11 (3)	2 (3)	13 (3)
Days of fever at admission	7 (5–14)	7 (5–10)	7 (5–14)
Reported antibiotic use	73 (20)	16 (24)	89 (21)
Provisional diagnosis			
Enteric fever	183 (51)	32 (48)	215 (50)
Fever/pyrexia of unknown origin	127 (35)	24 (36)	151 (35)
Dengue fever	22 (6)	6 (9)	28 (7)
Malaria^a^	11 (3)	6 (9)	17 (4)
Viral fever	6 (2)	3(4)	9 (2)
Urinary tract infection	3 (1)	0 (0)	3 (1)
Length of stay, d	7 (5–9)	6 (4–8)	6 (5–9)
Diagnosed with complication			
Hepatitis	24 (7)	2 (3)	26 (6)
Encephalopathy	11 (3)	0 (0)	11 (3)
Gastrointestinal bleeding	9 (3)	0 (0)	9 (2)
Renal impairment	7 (2)	3 (5)	10 (2)
Hemodynamic shock	7 (2)	0 (0)	7 (2)
Intestinal perforation	3 (<1)	0 (0)	3 (<1)
Myocarditis	1 (<1)	0 (0)	1 (<1)
Other complications	27 (7)	4 (6)	31 (7)

Data are no. (%) of patients or median (interquartile range).

Abbreviations: *S*. Paratyphi, *Salmonella enterica* subspecies *enterica* serovar Paratyphi; *S*. Typhi, *Salmonella enterica* subspecies *enterica* serovar Typhi.

^a^Significant difference between individuals infected with *S.* Typhi and those infected with *S.* Paratyphi (*P* < .05).

Among hospitalized patients with laboratory-confirmed infection, 76 (18%) had a diagnosis of at least 1 complication (68 [19%] infected with *S.* Typhi and 8 [12%] infected with *S*. Paratyphi); 16 (4%) had a diagnosis of >1 complication (15 [4%] infected with *S.* Typhi, compared with 1 [2%] infected with *S*. Paratyphi). The most commonly diagnosed complications were hepatitis (in 26 [6%]), encephalopathy (in 11 [3%]), gastrointestinal bleeding (in 9 [3%]), renal impairment (in 10 [2%]), and hemodynamic shock (in 7 [2%]), and intestinal perforation (in 3 [<1%]). A statistically longer median length of stay was observed in patients with any complications (7 days; IQR, 5–12 days), compared with patients without complications (6 days; IQR, 4–8 days; *P* = .004).

The case-fatality rate among hospitalized patients with laboratory-confirmed infection was 1.2% (5 of 429). Among fatal cases, the median age was 35 years (IQR, 18–52 years), and the sex was male in 80%. All 5 patients who died had ≥2 complications diagnosed, including encephalopathy (in 1), myocarditis (in 1), hemodynamic shock (in 4), hepatitis (in 2), renal impairment (in 3), and other complications (in 4).

Isolates among hospitalized laboratory-confirmed cases were predominantly susceptible to first-line drugs, with 418 of 428 (98%) susceptible to trimethoprim/sulfamethoxazole, 260 of 276 (94%) susceptible to ampicillin, and 266 of 272 (98%) susceptible to chloramphenicol. No isolates were observed to be multidrug resistant. The majority of *S.* Typhi and *S.* Paratyphi isolates were resistant to ciprofloxacin (352 of 427 [82%]) and nalidixic acid (414 of 425 [97%]); ceftriaxone resistance was reported in 4 isolates (1%) that were also resistant to ciprofloxacin. Azithromycin resistance was identified in 3 of 33 isolates (9%) tested. PGI reported significantly more isolates resistant to ampicillin than other hospitals (34% vs 0% at Medanta and Apollo and 2% at KMC and CMC; *P* < .0001), while Medanta and PGI reported a significantly smaller percentage of isolates resistant to ciprofloxacin than the other hospitals (49% and 16%, respectively, vs 90% at CMC and 99% at Apollo and KMC; *P* < .0001). Statistically significant differences were not observed in antimicrobial resistance patterns between *S.* Typhi and *S.* Paratyphi isolates, but the proportion of isolates with resistance to ciprofloxacin increased significantly from 2014 (159 of 212 [75%]) to 2015 (191 of 215 [89%]; *P* = .0005).

### Surgical Cases

Among the 94 patients with intestinal perforation who had clinical data, 16 (17%) had a provisional, final, or laboratory-confirmed diagnosis of enteric fever (all due to *S.* Typhi). Disease in 4 patients was laboratory confirmed (all due to *S.* Typhi), through either blood culture (in 3 [19%]) or histopathologic analysis (in 1 [6%]; Table 3). The median age of these 16 patients was 25.5 years (IQR, 19.5–33.5 years), and the sex in 88% was male. Of the 14 patients for whom data on the location of the perforation were available, perforations for all (100%) were in the ileum. Some symptoms, including nausea/vomiting, diarrhea, and weakness/malaise, were reported in similar proportions of patients with perforations and a provisional or confirmed diagnosis of enteric fever and all patients with laboratory-confirmed enteric fever. Compared with hospitalized patients with laboratory-confirmed disease, however, a significantly lower proportion had fever (75% vs 97%; *P* = .001), and significantly higher proportions had abdominal pain (75% vs 32%; *P* = .0003) or constipation (38% vs 3%; *P* < .0001).

**Table 3.** Clinical Presentation of Patients With Intestinal Perforation and a Provisional, Final, or Laboratory-Confirmed Diagnosis of Enteric Fever, All Hospital Sites, India, 2014–2015

Variable	Value^a^
Male sex	14 (88)
Age, y	25 (19.5–33.5)
Symptom	
Fever	12 (75)
Abdominal pain	12 (75)
Nausea/vomiting	9 (56)
Weakness/malaise	7 (44)
Constipation	6 (38)
Diarrhea	3 (19)
Prior antibiotic use	11 (69)
Length of stay, d	10 (6.5–12.5)
Complication	
Wound infection	3/14 (21)
Sepsis	2/14 (14)
Pulmonary complication	2/14 (14)
Shock	1/15 (7)
Other	3/11 (21)
Ileal perforation	14/14 (100)
Final outcome	
Discharged	14 (88)
Left against medical advice	1 (6)
Death	1 (6)

Data are no. or proportion (%) of patients or median (interquartile range).

^a^Data are for 16 patients, unless otherwise indicated.

## DISCUSSION

Our retrospective review of hospital records, spanning 2 years and including data from 5 hospitals across the country, indicates that enteric fever is still present in healthcare settings across India and predominately affects children and young adults. Our study captured 16 confirmed cases of ileal perforation and 16 laboratory-confirmed cases with multiple severe complications, including at least 5 fatalities. Prior to this study, enteric fever surveillance in India had been limited to mainly small single-hospital-based studies, leading to substantial knowledge gaps [3].

While published literature from studies conducted in South Asia over the past decade has consistently reported increasing resistance to fluoroquinolones, limiting the prescriptive use of drugs from this class, we also documented resistance to third-generation cephalosporins and macrolides, presently the treatments of choice in India [13, 20, 21]. These evolving antimicrobial resistance patterns should be carefully monitored in prospective studies. Increasing antimicrobial resistance in *S*. Typhi and *S*. Paratyphi isolates, such as that observed in the recent outbreak of extensively drug-resistant cases in Pakistan, also highlights the need for ongoing enteric fever surveillance and the potential benefits of rapid deployment of typhoid vaccines.

The age distribution we observed for all enteric fever cases is similar to results from other hospital-based studies in Asia [14, 22]. This is in contrast to community-based surveillance in previous studies, which reported a shift toward a higher prevalence in younger populations [4–6]. This difference may be explained by one of the limitations of hospital-based studies—the inability to control for healthcare-seeking behavior—which is also apparent in the sex-associated disparities in our data for both minor and adult populations. Previous hypotheses on sex-associated differences in healthcare-seeking behavior among children included parental decision to delay treatment and lower inclination to spend money on treatment for female children, compared with male children [23, 24]. These demographic data gaps in hospital-based surveillance are potentially controllable through a low-cost hybrid method using prospective health facility–based surveillance and household surveys to determine community healthcare utilization rates, which has been outlined by Luby et al [25]. Ascertaining the true burden of disease in the community will be crucial to accurately targeting high-risk populations for the new vaccine.

The age distribution in our study, and in previous studies, differs slightly by etiology (typhoid vs paratyphoid fever), although children and young adults bear the largest burden of both diseases [26–28]. The presentation of hospitalized patients with enteric fever resembled that described in previous studies, including a significantly higher rate of gastrointestinal symptoms in those infected with *S.* Typhi [29]. Our study found that infections with *S*. Typhi were more severe than infections with *S*. Paratyphi, including a higher proportion *S.* Typhi–infected patients admitted to the hospital (although the timing of hospitalization, whether before or after culture results were available, is not known), which is similar to other studies in Asia and Africa [30, 31]. However, some studies have found that the severity of *S.* Paratyphi infection is increasing and comparable with that of *S*. Typhi infection [32].

Typhoid-related intestinal perforations have been estimated to occur in 0.8%–39% of laboratory-confirmed cases, depending on the socioeconomic status of the country [33]. It can be difficult to isolate *S*. Typhi from persons with intestinal perforations, owing to the likelihood of antibiotic use before blood culture or surgery—only 49% of our surgical cases had blood culture performed. Future studies should consider using surgical surveillance to strengthen the link between perforation and enteric fever [34].

Last, our study looked for temporal patterns in the burden of enteric fever. The seasonal influence of monsoons on disease burden has been previously documented in tropical countries where enteric fever is endemic [35]. Of the 5 hospital sites, 3 (Apollo, PGI, and CMC) did not experience these seasonal patterns, suggesting that additional investigation is needed to understand the epidemiological and environmental factors that predominantly drive the disease burden in India. Of the 2 sites that experienced an increase in cases during the typical Indian monsoon months in 1 of 2 study years, 1 site (Medanta) experienced a large and prolonged increase in cases, by month. This notable occurrence highlights the need to develop and maintain surveillance systems that can analyze patterns of disease in real time to provide timely information for disease control efforts.

This retrospective study provides insights to inform the design of future surveillance systems for enteric fever in India, including information on the distribution of disease, disease presentation and outcomes, and antimicrobial resistance patterns. However, these study findings should be interpreted with several limitations in mind. First, since the study design was retrospective, the data are subject to the biases associated with any retrospective study, such as inconsistent case definitions and missing data (consider that one quarter of inpatient charts were not found for review). Second, the study hospitals did not have electronic clinical records, leading to limited analysis of clinical data of all cases identified in the laboratory. Third, surgical specimens from intestinal perforation cases were infrequently tested by histopathologic analysis or blood culture, leading to a gap in the data collected. Last, since these data are hospital based, information on enteric fever in the hospitals’ geographic area depends on care-seeking behavior.

South Asia has the highest estimated global burden of enteric fever morbidity and mortality; however, current surveillance capabilities have not permitted an accurate estimate of the full spectrum of the impact that enteric fever has on the region. Further elucidating the link between severe complications and typhoid can also provide information on the potential benefits of typhoid vaccination campaigns. As the newly World Health Organization–recommended TCV has been shown to be 50%–87% efficacious, most if not all of these severe cases and deaths could be preventable with broad use of the vaccine [36]. In addition, broad implementation of TCV may help reduce transmission of typhoid, including resistant strains. In addition to describing the severity of disease and presence of antimicrobial resistance, national data on the burden of typhoid fever should incite Indian policymakers to consider including TCV in their immunization programs. Evidence-based decision-making using these types of regional-level data is crucial to reducing the impact of enteric fever in countries of endemicity.

## Notes

***Acknowledgments.*** We thank our colleagues Jacob John, Balaji Veeraraghavan, Pallab Ray, Vikas Goutam, Sharmila Sengupta, Amarjeet Kaur, Ujjwayini Khan Roy, Soma Dutta, K. E. Vandana, and Yasha Mukim, for their invaluable contributions to this project.

***Financial support.*** This work was supported by the Bill and Melinda Gates Foundation (grant OPP11130007).

***Supplement sponsorship.*** This article is part of the supplement “Surveillance for Enteric Fever in Asia Project,” sponsored by the Sabin Vaccine Institute.

***Potential conflicts of interest.*** All authors: No reported conflicts of interest. All authors have submitted the ICMJE Form for Disclosure of Potential Conflicts of Interest. Conflicts that the editors consider relevant to the content of the manuscript have been disclosed.
